# Factors Associated with Long-Term Control of Type 2 Diabetes Mellitus

**DOI:** 10.1155/2016/2109542

**Published:** 2016-12-20

**Authors:** Mohammed Badedi, Yahiya Solan, Hussain Darraj, Abdullah Sabai, Mohamed Mahfouz, Saleh Alamodi, Abdullah Alsabaani

**Affiliations:** ^1^Public Health Administration, Jazan Health Affairs, Ministry of Health, Jazan, Saudi Arabia; ^2^Diabetes Center, Jazan Health Affairs, Jazan, Saudi Arabia; ^3^Jazan Health Affairs, Ministry of Health, Jazan, Saudi Arabia; ^4^Faculty of Medicine, Jazan University, Jazan, Saudi Arabia; ^5^Jazan General Directorate of Education, Ministry of Education, Jazan, Saudi Arabia; ^6^College of Medicine, King Khalid University, Abha, Saudi Arabia

## Abstract

*Aims.* This study assessed factors associated with glycemic control among Saudi patients with Type 2 diabetes mellitus (T2DM).* Methods.* We conducted an analytical cross-sectional study, which included a random sample of 288 patients with T2DM proportional to the diabetes population of each primary health care center in Jazan city, Kingdom of Saudi Arabia.* Results.* More than two-thirds (74%) of patients had poor glycemic control. Lack of education, polypharmacy, and duration of diabetes ≥ 7 years were significantly associated with higher glycated hemoglobin (HbA1c). Moreover, patients who were smoker or divorced were significantly more likely to have higher HbA1c. The patients who did not comply with diet or take their medications as prescribed had poor glycemic control. The study found lower HbA1c levels among patients who received family support or had close relationship with their physicians. Similarly, knowledgeable patients towards diabetes or those with greater confidence in ability to manage self-care behaviors had a lower HbA1c. In contrast, risk factors such as depression or stress were significantly correlated with poorer glycemic control.* Conclusion.* The majority of T2DM patients had poor glycemic control. The study identified several factors associated with glycemic control. Effective and tailored interventions are needed to mitigate exposure to these risk factors. This would improve glycemic control and reduce the risks inherent to diabetes complications.

## 1. Introduction

 The prevalence of diabetes in Saudi Arabia is estimated to be 20.5% among people aged between 20 and 79 years in 2014 [1]. Recently, in the southwestern region of Saudi Arabia (Jazan region), Bani reported that the overall prevalence of diabetes mellitus was 12.3% [2]. The impact of diabetes is reflected not only in the increasing number of people with diabetes but also in the growing number of premature deaths caused by diabetes and its complications. Many studies have emphasized that glycemic control (HbA1c ≤ 7%) reduces the risk of complications [3–7]. Despite the importance of achieving the recommended glycemic control, the majority of patients with diabetes have a poor glycemic control [8]. Thus, it is important to identify factors and barriers to improving glycemic control. However, little is actually known about the factors that are associated with glycemic control among patients with diabetes. While several studies have been conducted to explore these factors, they were primarily conducted in western countries [9–21]. These factors might differ from one population to another based on differences in religion, culture, behavior, education, and income. To the best of our knowledge, this is the first study that has been carried out on patinets with T2DM to identfy factors related to glycemic control in the Jazan region of Saudi Arabia. Due to population and demographic differences, further studies are needed to explore these factors among Jazan's population. This study assessed the factors and barriers associated with glycemic control over the last three months. Moreover, we estimated the prevalence of good and poor glycemic control among patients with T2DM in Jazan city.

## 2. Methods

### 2.1. Participants

The study involved an analytical cross-sectional approach. A stratified cluster sampling technique was used to ensure that the sample was representative of the target population. In Jazan city, all patients with T2DM who had been diagnosed and registered in the primary health care centers' registries were divided into two male and female strata. A random sample was then selected proportionally to the diabetes population of each primary health care center.

### 2.2. Data Collection Instruments and Measurements

Face-to-face interviews were held using a valid questionnaire created by an interdisciplinary team from the Carver College of Medicine, the College of Pharmacy, and the College of Public Health at the University of Iowa [10]. The questionnaire included sociodemographic variables, health risk variables (smoking, duration of diabetes, and other comorbidities), adherence to self-care behaviors (following meal plan, taking medications, exercising regularly, and testing blood glucose at home), and anthropometric variables (blood pressure, body mass index, and lipid profile). In addition, the questionnaire assessed barriers to self-care behaviors, family support, physician-patient communication, mental and physical health, knowledge towards diabetes, confidence in ability to manage self-care behaviors, and motivation to do a better job in self-care behaviors.

In addition, a question regarding the presence of stressful life events was added. Stressful life events were measured using a validated stress scale developed by psychiatrists Holmes and Rahe in 1967 [22]. The body mass index was calculated as weight in kilograms divided by height squared [23]. The blood pressure was measured using standardized mercury sphygmomanometer EN 1060. It was measured while the patient was seated, and it was repeated after 5 minutes of rest [24]. The lipid profile was analyzed including cholesterol, high-density lipoprotein (HDL), low-density lipoprotein (LDL), and triglyceride [24].

### 2.3. Questionnaire Translation and Validation

The questionnaire was translated from English into Arabic. The steps of translation were based on the guidelines of translation and cross-cultural adaptation by Beaton et al. [25]. Face validity, internal consistency, and test-retest reliability were assessed to validate the Arabic-language version. Cronbach's Alpha values for the items addressing barriers to self-care behaviors, depression, and stressful life events variables were 0.81, 0.83, and 0.74, respectively.

### 2.4. Biomedical Investigation Tools

The HbA1c was measured for all patients in one laboratory using the same measurement tool (DCA Vantage Analyzer, Siemens, UK). A registered nurse collected blood samples into EDTA-K2 tubes. The blood samples for lipid profiles were drawn after fasting for at least 14 hours and collected in serum separator tubes. The samples were analyzed using UniCel DxC 600 Synchron Clinical Systems, Beckman Coulter, USA.

### 2.5. Data Analysis

HbA1c is the main dependent and outcome variable, and it was tested for an association with other potentially independent variables. Dichotomous groups were created for all variables. The HbA1c distribution was positively skewed even after transforming by square root and log⁡10. Due to this violation of the normality, the difference between groups was explored using nonparametric tests (Mann–Whitney *U* test and Kruskal-Wallis ANOVA test). A Bonferroni adjustment was computed to determine the alpha values to control Type 1 error. The Mann–Whitney *U* test was used to estimate the actual differences among groups derived through the Kruskal-Wallis ANOVA test. Spearman's rho was used to explore the relationship between HbA1c outcome and ordinal predictors (Likert scales). A logistic regression model was used to identify predictors correlated with HbA1c. The logistic regression assumptions (multicollinearity) were checked for high intercorrelations among predictors. A chi-squared test was used to assess the significant relationship between categorical variables. A *P* value of >0.05 was considered to be statistically significant.

## 3. Result

This study included 288 Saudi patients with T2DM. The response rate was 93.8%. Of the total respondents, 74% had poor glycemic control (HbA1c < 7%). The median of HbA1c for the study subjects was 8.7 with an interquartile range (IQR) of 7 to 10.3.

### 3.1. Sociodemographic and Health Risks with HbA1c

The respondents were between 28 and 83 years old with a mean (SD) of 54.58 (10.9). The median duration of diabetes was seven years, and it ranged between 4 and 14 years. Higher HbA1c levels were significantly associated with younger age (*P* < 0.01). There were no associations between HbA1c levels and sex or occupation. Divorced subjects had a higher HbA1c while married subjects had a lower HbA1c. Furthermore, lower HbA1c was significantly associated with higher educational level. Regarding the health risk variables, smokers were significantly more likely to have higher HbA1c compared to nonsmokers. Higher HbA1c levels were significantly correlated with irritable bowel syndrome (IBS) and longer duration of diabetes (Table 1).

### 3.2. Self-Care Behavior's Performance and HbA1c

The majority of patients (80.6%) did not follow their recommended meal plan, while two-thirds (69.1%) of patients adhered to their prescribed medications. The lower HbA1c levels were associated with higher adherence to medication or following meal plans. However, HbA1c levels were not associated with higher adherence to regular exercise or testing blood glucose at home (Table 2).

The patients who followed their meal plan and took their medications as prescribed had lower HbA1c, as did persons who reported high adherence with meal plans and exercise. Furthermore, patients who followed meal plans, took medication as prescribed, exercised regularly, and did home blood testing recorded good glycemic control. Patients who never discussed their diet plan with their dietitians or who were on >4 medications had higher HbA1c (*P* < 0.001) (Table 2).

### 3.3. Barriers of Self-Care Behavior and HbA1c

Seven to fourteen barriers were assessed for the four self-care behaviors. Barriers were rated from one to five with five indicating a barrier to greater effects on the specified self-care behavior. The barrier “too busy” was significantly associated with higher HbA1c for all self-care behaviors. Other barriers and their significant correlations with higher HbA1c for each self-care behavior are shown in Table 3.

### 3.4. Family Support and Physician-Patient Relationship

Most patients who received adequate support from their family and friends and had a good relationship with their physicians had lower HbA1c (Table 4). Transportation difficulties as well as barriers such as “forget” and “busy” hindered a significant percentage of the respondents from honoring doctors' appointment and regularly attending the clinics.

### 3.5. Knowledge towards Diabetes and Attitude to Self-Care Behavior

Patients who were knowledgeable towards diabetes had a lower HbA1c. Greater confidence in the ability to manage self-care behaviors was also significantly correlated with lower HbA1c. Patients with good glycemic control were extremely motivated to do a better job of diabetes self-care (Table 4).

### 3.6. Physical Health, Depression, and Stressful Life Events and HbA1c

The median for general mental health perception score was 49.1 and the interquartile range (IQR) was from 42.9 to 57.3. Poor general mental health perceptions led to high HbA1c level. Patients with a high PHQ-9 depression score had a high HbA1c level. The PHQ-9 median score was 2 (IQR: 0, 10). Higher stressful life events scores were significantly correlated with higher HbA1c levels. The median of the stressful life events score was 38 with a range of 0 to 102 (Table 4).

### 3.7. Anthropometric and Biomedical Variables and HbA1c

The respondents' body mass index (BMI) ranged from 26.4 to 33.7 (median = 29.4). Patients with poor glycemic control had a high BMI. However, the study did not observe any significant association between HbA1c and hypertension. In the lipid profile, the mean for blood cholesterol was 191.4 (SD = 40.7) with a range of 98.9 to 296 mg/dL. Patients were more likely to have poorly controlled blood glucose among those with high blood cholesterol level, high LDL, and high triglyceride levels. Patients with good glycemic control had HDL ≥ 40 for men and ≥50 for women (Table 4).

### 3.8. Logistic Regression Analysis of Factors Associated with HbA1c

Variables in the regression model included not taking medication (OR = 4.06, *P* = 0.013), number of medications (OR = 7.49, *P* > 0.005), extended duration of diabetes (OR = 4.64, *P* = 0.001), and low confidence in the ability to control diabetes. These variables were significantly associated with increased odds of being poorly glycemic controlled (Table 5).

## 4. Discussion

This study assessed the adherence and barriers to diabetes control, self-care behaviors, and their association with HbA1c levels among Saudi patients with T2DM. We found that 74.3% of the respondents had poor glycemic control (HbA1c < 7%). This was significantly similar to other Saudi populations. Several studies reported similar prevalence of poor glycemic control (73%, 76%, and 79.4%) [26–28]. Compared to other countries, the proportion of poor glycemic control among patients with diabetes was 66.7% in Kuwait and 69% in the UAE [29, 30]. In Jordon, only 34.9% of patients with diabetes reached the target level of glycemic control [11].

This study also showed that sociodemographic factors influenced the HbA1c levels. For example, younger and less educated participants recorded higher HbA1c levels than older and more informed counterparts. These findings are consistent with other studies [9–13]. This study also found that those with lower HbA1c complied with the recommended diabetes diet and took their medications as directed by physicians. On the other hand, patients with poor glycemic control were often on several medications and had lived with diabetes for a longer duration. These findings agree with other studies [9–12, 19]. It is known that poor glycemic control is significantly associated with a longer duration of diabetes and polypharmacy. Diabetes is a progressive disease and as glucose levels rise, more drugs are required to achieve control. Moreover, a longer duration of diabetes is known to be associated with poor glycemic control, and this could be explained by progressive impairment of insulin secretion over time because of beta cell failure. This makes the response to diet alone or oral agents unlikely [4].

This study also showed that physical activity did not play a significant role in glycemic control. Such results contradict several past studies that have linked physical inactivity to high glucose levels [10–12, 16]. This is due to the fact that Jazan city is hot, and this discourages diabetics from exercising. However, the participants who combined exercise with appropriate diet did achieve better glycemic control. Therefore, we concluded that the physical activity could only lead to the targeted glycemic levels when accompanied by healthy eating habits.

Similarly, participants who discussed their diet plan with their dietitians recorded higher HbA1c. This refers to the health system of PHCC in Jazan, which usually promotes the appointment between dietitians and patients with poor glycemic control to improve diet compliance. Better physician-patient communication was associated with better glycemic control. Good physician relationships with patients play an important role in achieving adherence to diabetes care plans. Similarly, family and friends who provided adequate support to patients with diabetes played a critical role in improving their glycemic control. An individual's environment is critical to maintaining accepted glycemic levels. These findings concur with other studies that found that family support and close relationships with a physician improve HbA1c level [10].

Diabetics need proper knowledge to improve their condition and prevent complications. The more knowledgeable the patients are towards diabetes, the more compliant they are to the care plan. As a result, patients with poor glycemic control lacked appropriate knowledge on how to improve glycemic control. Other studies have shown similar results [10, 11, 17, 20].

In addition, this study has shown that risk factors such as depression, poor general health, high-stress levels, and obesity result in poor glycemic control. Prior studies have shown similar findings [10, 11, 16, 18]. Other researchers have also shown that depressive symptoms adversely affect metabolic activities, which in turn inhibit the body from using the excess glucose in the blood, which causes insulin resistance. This deteriorates glycemic control [31]. At the same time, stressed patients with diabetes are more likely to lead unhealthy lifestyles than their peers who enjoy social support from family members and friends [32].

## 5. Conclusion

The percentage of patients with poor glycemic control and noncompliance to self-care behaviors was high in Jazan city. Therefore, medical practitioners should incorporate these essential factors into diabetes care to improve glycemic control and prevent diabetes complications. The study revealed that Saudi patients with T2DM were at greater risks than other populations because they are reluctant to adhere to the prescribed self-care behaviors. However, the stakeholders can successfully address these issues by creating regular educational forums and awareness programs that focus on these essential self-care areas. The educators should focus on critical factors such as building positive attitudes and the benefits of monitoring the glycemic levels. The study demonstrated that health experts should not only develop relationships with their patients but also encourage their clients to be proactive in managing their conditions. This includes gathering information, sharing their experiences with all the relevant stakeholders, and incorporating best practices into their self-care plans. As such, patients with diabetes will achieve better treatment outcomes.

## Figures and Tables

**Table 1:** Sociodemographic and health risk factors.

Variable	Categories	*n* (%)	HbA1c	*P*
Age (year)	28–49	*n* = 87 (30.2%)	9	0.011
	50–64	*n* = 148 (51.4%)	8.7	
	64–83	*n* = 53 (18.4%)	7.7	

Sex	Male	*n* = 145 (50.3%)	8.5	0.083
	Female	*n* = 143 (49.7%)	8.9	

Marital status	Divorced	*n* = 7 (2.4%)	11.5	0.005
	Single	*n* = 16 (5.6%)	9.5	
	Widowed	*n* = 36 (12.5%)	9.4	
	Married	*n* = 229 (79.5%)	8.5	

Education level	Illiterate	*n* = 36 (12.5%)	9.2	0.032
	Read and write	*n* = 33 (11.5%)	9.1	
	Elementary school level	*n* = 41 (14.2%)	8.9	
	Intermediate school level	*n* = 42 (14.6%)	8.8	
	Secondary school level	*n* = 57 (19.8%)	8.2	
	University level	*n* = 79 (29.4%)	8.1	

Occupation	Unemployed	*n* = 8 (2.1%)	8.8	0.691
	Employed	*n* = 105 (36.5%)	8.3	
	Retired	*n* = 67 (23.3%)	8.7	
	Homemaker	*n* = 103 (35.8%)	8.9	
	Businessman	*n* = 4 (1.4%)	8.9	
	Disabled	*n* = 3 (1%)	7.6	

Smoking history	Smoker	*n* = 63 (21.9%)	9.4	0.031
	Ex-smoker	*n* = 2 (0.7%)	8.6	
	Nonsmoker	*n* = 223 (77.4%)	8.5	

Duration of diabetes (year)	≥7	*n* = 166 (42.4%)	9.1	>0.001
	>7	*n* = 122 (57.6%)	7.5	

Other chronic diseases or diabetes complications	Irritable bowel syndrome (IBS)	*n* = 9 (3.1%)	11.5	0.020
	Hypertension (HTN)	*n* = 162 (56.2)	8.8	
	Asthma	*n* = 6 (2.1%)	8.8	
	No other chronic disease or diabetes complications	*n* = 111 (38.6%)	8.5	

**Table 2:** Self-care behavior's adherence and HbA1c.

Variable	Categories	*n* (%)	HbA1c (%)	*P*
Following a meal plan	Low adherence	*n* = 232 (80.6%)	9.0	>0.001
	High adherence	*n* = 56 (19.4%)	7.3	

Taking medications	Low adherence	*n* = 89 (30.9%)	9.2	0.001
	High adherence	*n* = 199 (69.1%)	8.2	

Exercising	Low adherence	*n* = 121 (42%)	8.8	0.310
	High adherence	*n* = 167 (58%)	8.6	

Testing blood glucose	Low adherence	*n* = 146 (50.7%)	8.9	0.301
	High adherence	*n* = 142 (49.3%)	8.6	

Following a meal plan and taking medication	Low adherence	*n* = 80 (27.8%)	9.4	>0.001
	High adherence	*n* = 47 (16.3%)	7.0	

Following a meal plan and exercising regularly	Low adherence	*n* = 105 (36.5%)	9.0	>0.001
	High adherence	*n* = 40 (13.9%)	7.4	

Following a meal plan, taking medication, exercising, testing blood glucose	Low adherence	*n* = 37 (12.8%)	10.1	>0.001
	High adherence	*n* = 26 (9%)	6.9	

Number of medications	<4	*n* = 136 (47.2%)	9.5	0.001
	≤4	*n* = 152 (52.8%)	7.4	

Treatment modalities	Oral antidiabetic agents alone	*n* = 229 (79.5%)	8.7	0.740
	Oral antidiabetic agents and insulin	*n* = 59 (20.5%)	8.7	

Medication and treatment modalities	Low medication adherence	*n* = 26 (9%)	9.5	0.001
	Oral antidiabetic agents with insulin	*n* = 60 (20.8%)	9.2	
	Low medication adherence—oral antidiabetic agents alone	*n* = 169 (58.7%)	8.2	
	High medication adherence—oral antidiabetic agents alone	*n* = 33 (11.5%)	8.1	
	High medication adherence—oral antidiabetic agents with insulin			

**Table 3:** Correlations between barriers of self-care behaviors and HbA1c.

Barrier was a significant problem	Following a meal plan (*r*)	Taking medications (*r*)	Exercising regularly (*r*)	Monitoring blood glucose (*r*)
Too busy and care about other things	0.22^*∗∗*^	0.21^*∗∗*^	0.13^*∗*^	0.30^*∗∗*^
Hassle	0.26^*∗∗*^	0.04	0.25^*∗∗*^	0.10
Forgot	0.08	0.37^*∗∗*^	0.22^*∗∗*^	0.24^*∗∗*^
Don't believe	0.06	0.09	0.05	0.06
Don't understand	0.13^*∗*^	0.16^*∗∗*^	0.19^*∗∗*^	0.27^*∗∗*^
Don't like	0.05	0.13^*∗*^	0.20^*∗∗*^	0.05
Depression interferes	0.12^*∗*^	0.12^*∗*^	0.16^*∗∗*^	0.03
Sometimes don't have	N/A	0.17^*∗∗*^	N/A	0.19^*∗*^
Concerned about side effects	N/A	0.18^*∗∗*^	N/A	N/A
Cannot exercise (disabled)	N/A	N/A	0.12^*∗*^	N/A
Bad weather	N/A	N/A	0.21^*∗∗*^	N/A
Shortness of breath	N/A	N/A	0.09	N/A
Knee pain	N/A	N/A	0.31^*∗∗*^	N/A
Hurts	N/A	N/A	N/A	0.15^*∗*^

^*∗*^ *P* ≤ 0.05; ^*∗∗*^*P* ≤ 0.01 or ≤ 0.001; N/A means the barrier was not related to the self-care behavior.

**Table 4:** Anthropometrics and HbA1c levels.

Variable	Categories	*n* (%)	HbA1c	*P*
Family provides help and support	Lesser extent (a little)	*n* = 76 (26.4%)	9.4	0.002
	Greater extent (a lot)	*n* = 212 (73.6%)	8.4	

Physician-patient relationship	Lesser extent (seldom)	*n* = 41 (14.2%)	10.6	>0.001
	Greater extent (often)	*n* = 247 (85.8%)	8.5	

Knowledge towards diabetes	Lesser extent	*n* = 136 (47.2%)	8.9	0.020
	Greater extent	*n* = 152 (52.8%)	8.5	

Confidence in ability to manage self-care behaviors	Not confident	*n* = 159 (55.2%)	8.9	0.001
	Confident	*n* = 129 (44.8%)	8.5	

Physical health	<40	*n* = 57 (19.8%)	11.8	>0.001
	40–50	*n* = 93 (32.3%)	9.12	
	>50	*n* = 138 (47.9%)	7.50	

Depression	Major depression	*n* = 41 (14.2%)	11.3	>0.001
	Atypical depression	*n* = 63 (21.9%)	8.70	
	No depression	*n* = 184 (63.9%)	7.85	

Stressful life events	High risk <300	*n* = 24 (8.3%)	11.8	>0.001
	Moderate risk 150–300	*n* = 45 (15.6%)	8.90	
	Low risk >150	*n* = 219 (76%)	8.00	

Blood pressure (BP) (mmHg)	High blood pressure	*n* = 127 (44.1%)	9.0	0.073
	Normal blood pressure	*n* = 161 (55.9%)	8.5	

Body mass index (BMI) (kg/m^2^)	Obese	*n* = 134 (46.5%)	8.9	0.01
	Overweight	*n* = 107 (37.2%)	8.7	
	Normal weight	*n* = 44 (15.3%)	7.9	
	Underweight	*n* = 3 (1%)	6.3	

Cholesterol (mg/dL)	Blood cholesterol ≥ 200	*n* = 117 (40.6%)	9.2	>0.001
	Blood cholesterol > 200	*n* = 171 (59.4%)	8.1	

High-density lipoprotein (HDL) (mg/dL), male	Low HDL > 40	*n* = 80 (55.2%)	9.1	>0.001
	High HDL ≥ 40	*n* = 65 (44.8%)	7.6	

High-density lipoprotein (HDL) (mg/dL), female	Low HDL > 50	*n* = 94 (65.7%)	9.1	0.027
	High HDL ≥ 50	*n* = 49 (34.3%)	7.8	

Low-density lipoprotein (LDL) (mg/dL)	High LDL ≥ 100	*n* = 198 (68.8%)	8.8	0.026
	Low LDL > 100	*n* = 90 (31.2%)	8.2	

Triglyceride (TG) (mg/dL)	High TG ≥ 150	*n* = 116 (40.3%)	9.1	>0.01
	Low TG > 150	*n* = 172 (59.7%)	8.4	

**Table 5:** Regression model for factors associated with HbA1c.

Variable	Categories	OR (95% confidence interval)	*P*
Taking medication	Low adherence	4.06 (1.34, 12.27)	0.013
	High adherence		
Number of medications	<4	7.49 (3.45, 16.26)	>0.005
	≤4		
Duration of diabetes (year)	≥7	4.64 (1.85, 11.67)	0.001
	>7		
Confidence in ability to manage self-care behaviors	Not confident	4.01 (1.52, 10.63)	0.005
	Confident		
