# Erratum to “Factors Associated with Long-Term Control of Type 2 Diabetes Mellitus”

**DOI:** 10.1155/2019/8756138

**Published:** 2019-05-06

**Authors:** Mohammed Badedi, Yahiya Solan, Hussain Darraj, Abdullah Sabai, Mohamed Mahfouz, Saleh Alamodi, Abdullah Alsabaani

**Affiliations:** ^1^Public Health Administration, Jazan Health Affairs, Ministry of Health, Jazan, Saudi Arabia; ^2^Diabetes Center, Jazan Health Affairs, Jazan, Saudi Arabia; ^3^Jazan Health Affairs, Ministry of Health, Jazan, Saudi Arabia; ^4^Faculty of Medicine, Jazan University, Jazan, Saudi Arabia; ^5^Jazan General Directorate of Education, Ministry of Education, Jazan, Saudi Arabia; ^6^College of Medicine, King Khalid University, Abha, Saudi Arabia

In the article titled “Factors Associated with Long-Term Control of Type 2 Diabetes Mellitus” [1], the less and greater than signs were reversed in the tables and the text. In addition, there were minor errors in Introduction and the format of Table 2.

1. In Introduction, there was a minor spelling error where the text reading “To the best of our knowledge, this is the first study that has been carried out on patinets with T2DM to identfy factors related to glycemic control in the Jazan region of Saudi Arabia” should be corrected to “To the best of our knowledge, this is the first study that has been carried out on patients with T2DM to identify factors related to glycemic control in the Jazan region of Saudi Arabia.”
2. The last sentence in the “Data Analysis” subsection in the Methods reading “A *P* value of >0.05 was considered to be statistically significant” should be corrected to “A *P* value of < 0.05 was considered to be statistically significant.”
3. In the first paragraph of the “Result” section, the sentence reading “Of the total respondents, 74% had poor glycemic control (HbA1c < 7%)” should be corrected to “Of the total respondents, 74% had poor glycemic control (HbA1c > 7%).”
4. In Table 1, the category “Duration of diabetes (year)” included reversed less and greater than signs. The corrected table is as shown below.
5. In Table 2, the less and greater than signs were reversed in many categories and the format of the category “Medication and treatment modalities” should be updated for clarity. The corrected table is as shown below.
6. In the subsection titled “Logistic Regression Analysis of Factors Associated with HbA1c” in the “Result” section, the text reading “Variables in the regression model included not taking medication (OR = 4.06, *P* = 0.013), number of medications (OR = 7.49, *P* > 0.005), extended duration of diabetes (OR = 4.64, *P* = 0.001), and low confidence in the ability to control diabetes” should be corrected to “Variables in the regression model included not taking medication (OR = 4.06, *P* = 0.013), number of medications (OR = 7.49, *P* < 0.005), extended duration of diabetes (OR = 4.64, *P* = 0.001), and low confidence in the ability to control diabetes.”
7. In Table 4, the less and greater than signs were reversed in many categories. The corrected table is as shown below.
8. In Table 5, the less and greater than signs were reversed in many categories. The corrected table is as shown below.

## Figures and Tables

**Table 1:** Sociodemographic and health risk factors.

Variable	Categories	*n* (%)	HbA1c	*P*
Age (year)	28-49	*n* = 87 (30.2%)	9	.011
	50-64	*n* = 148 (51.4%)	8.7	
	64-83	*n* = 53 (18.4%)	7.7	
Sex	Male	*n* = 145 (50.3%)	8.5	.083
	Female	*n* = 143 (49.7%)	8.9	
Marital status	Divorced	*n* = 7 (2.4%)	11.5	.005
	Single	*n* = 16 (5.6%)	9.5	
	Widowed	*n* = 36 (12.5%)	9.4	
	Married	*n* = 229 (79.5%)	8.5	
Education level	Illiterate	*n* = 36 (12.5%)	9.2	.032
	Read and write	*n* = 33 (11.5%)	9.1	
	Elementary school level	*n* = 41 (14.2%)	8.9	
	Intermediate school level	*n* = 42 (14.6%)	8.8	
	Secondary school level	*n* = 57 (19.8%)	8.2	
	University level	*n* = 79 (29.4%)	8.1	
Occupation	Unemployed	*n* = 8 (2.1%)	8.8	.691
	Employed	*n* = 105 (36.5%)	8.3	
	Retired	*n* = 67 (23.3%)	8.7	
	Homemaker	*n* = 103 (35.8%)	8.9	
	Businessman	*n* = 4 (1.4%)	8.9	
	Disabled	*n* = 3 (1%)	7.6	
Smoking history	Smoker	*n* = 63 (21.9%)	9.4	.031
	Ex-smoker	*n* = 2 (0.7%)	8.6	
	Nonsmoker	*n* = 223 (77.4%)	8.5	
Duration of diabetes (year)	≥7	*n* = 166 (42.4%)	9.1	<.001
	<7	*n* = 122 (57.6%)	7.5	
Other chronic diseases or diabetes complications	Irritable bowel syndrome (IBS)	*n* = 9 (3.1%)	11.5	.020
	Hypertension (HTN)	*n* = 162 (56.2)	8.8	
	Asthma	*n* = 6 (2.1%)	8.8	
	No other chronic disease or diabetes complications	*n* = 111 (38.6%)	8.5	

**Table 2:** Self-care behavior's adherence and HbA1c.

Variable	Categories	*n* (%)	HbA1c (%)	*P*
Following a meal plan	Low adherence	*n* = 232 (80.6%)	9.0	<.001
	High adherence	*n* = 56 (19.4%)	7.3	
Taking medications	Low adherence	*n* = 89 (30.9%)	9.2	.001
	High adherence	*n* = 199 (69.1%)	8.2	
Exercising	Low adherence	*n* = 121 (42%)	8.8	.310
	High adherence	*n* = 167 (58%)	8.6	
Testing blood glucose	Low adherence	*n* = 146 (50.7%)	8.9	.301
	High adherence	*n* = 142 (49.3%)	8.6	
Following a meal plan and taking medication	Low adherence	*n* = 80 (27.8%)	9.4	<.001
	High adherence	*n* = 47 (16.3%)	7.0	
Following a meal plan and exercising regularly	Low adherence	*n* = 105 (36.5%)	9.0	<.001
	High adherence	*n* = 40 (13.9%)	7.4	
Following a meal plan, taking medication, exercising, and testing blood glucose	Low adherence	*n* = 37 (12.8%)	10.1	<.001
	High adherence	*n* = 26 (9%)	6.9	
Number of medications	>4	*n* = 136 (47.2%)	9.5	.001
	≤4	*n* = 152 (52.8%)	7.4	
Treatment modalities	Oral antidiabetic agents alone	*n* = 229 (79.5%)	8.7	.740
	Oral antidiabetic agents and insulin	*n* = 59 (20.5%)	8.7	
Medication and treatment modalities	Low medication adherence—oral antidiabetic agents with insulin	*n* = 26 (9%)	9.5	.001
	Low medication adherence—oral antidiabetic agents alone	*n* = 60 (20.8%)	9.2	
	High medication adherence—oral antidiabetic agents alone	*n* = 169 (58.7%)	8.2	
	High medication adherence—oral antidiabetic agents with insulin	*n* = 33 (11.5%)	8.1	

**Table 3:** Anthropometrics and HbA1c levels.

Variable	Categories	*n* (%)	HbA1c	*P*
Family provides help and support	Lesser extent (a little)	*n* = 76 (26.4%)	9.4	.002
	Greater extent (a lot)	*n* = 212 (73.6%)	8.4	
Physician-patient relationship	Lesser extent (seldom)	*n* = 41 (14.2%)	10.6	<.001
	Greater extent (often)	*n* = 247 (85.8%)	8.5	
Knowledge towards diabetes	Lesser extent	*n* = 136 (47.2%)	8.9	.020
	Greater extent	*n* = 152 (52.8%)	8.5	
Confidence in ability to manage self-care behaviors	Not confident	*n* = 159 (55.2%)	8.9	.001
	Confident	*n* = 129 (44.8%)	8.5	
Physical health	<40	*n* = 57 (19.8%)	11.8	<.001
	40-50	*n* = 93 (32.3%)	9.12	
	>50	*n* = 138 (47.9%)	7.50	
Depression	Major depression	*n* = 41 (14.2%)	11.3	<.001
	Atypical depression	*n* = 63 (21.9%)	8.70	
	No depression	*n* = 184 (63.9%)	7.85	
Stressful life events	High risk >300	*n* = 24 (8.3%)	11.8	<.001
	Moderate risk 150-300	*n* = 45 (15.6%)	8.90	
	Low risk <150	*n* = 219 (76%)	8.00	
Blood pressure (BP) (mmHg)	High blood pressure	*n* = 127 (44.1%)	9.0	.073
	Normal blood pressure	*n* = 161 (55.9%)	8.5	
Body mass index (BMI) (kg/m^2^)	Obese	*n* = 134 (46.5%)	8.9	.01
	Overweight	*n* = 107 (37.2%)	8.7	
	Normal weight	*n* = 44 (15.3%)	7.9	
	Underweight	*n* = 3 (1%)	6.3	
Cholesterol (mg/dl)	Blood cholesterol ≥ 200	*n* = 117 (40.6%)	9.2	<.001
	Blood cholesterol < 200	*n* = 171 (59.4%)	8.1	
High-density lipoprotein (HDL) (mg/dl), male	Low HDL < 40	*n* = 80 (55.2%)	9.1	<.001
	High HDL ≥ 40	*n* = 65 (44.8%)	7.6	
High-density lipoprotein (HDL) (mg/dl), female	Low HDL < 50	*n* = 94 (65.7%)	9.1	.027
	High HDL ≥ 50	*n* = 49 (34.3%)	7.8	
Low-density lipoprotein (LDL) (mg/dl)	High LDL ≥ 100	*n* = 198 (68.8%)	8.8	.026
	Low LDL < 100	*n* = 90 (31.2%)	8.2	
Triglyceride (TG) (mg/dl)	High TG ≥ 150	*n* = 116 (40.3%)	9.1	<.01
	Low TG < 150	*n* = 172 (59.7%)	8.4	

**Table 4:** Regression model for factors associated with HbA1c.

Variable	Categories	OR (95% confidence interval)	*P*
Taking medication	Low adherence	4.06 (1.34, 12.27)	.013
	High adherence		
Number of medications	>4	7.49 (3.45, 16.26)	<.005
	≤4		
Duration of diabetes (year)	≥7	4.64 (1.85, 11.67)	.001
	<7		
Confidence in ability to manage self-care behaviors	Not confident	4.01 (1.52, 10.63)	.005
	Confident		
